# Statistical analysis of the maternal death rate at the Ebonyi State University Teaching Hospital, Abakaliki, for the year ending 31 December 2007

**DOI:** 10.4102/phcfm.v1i1.84

**Published:** 2009-09-15

**Authors:** Uchechukwu M. Okeh

**Affiliations:** 1 Industrial Mathematics and Applied Statistics, Ebonyi State University, Nigeria

**Keywords:** maternal deaths, Abakaliki, booked patients, unbooked patients, parity

## Abstract

**Background:**

The maternal mortality rate in developing countries, such as Nigeria, remains relatively high, with the causes being multidimensional. The unbooked primigravidae with severe pre-eclampsia/eclampsia constitute a high risk group.

**Method:**

The data from the case notes of all the maternal deaths that occurred at the Ebonyi State University Teaching Hospital, Abakaliki, between 1 January and 31 December 2007 form the basis of this study. The case notes relating to all such deaths were stored in the office of the Head of the Department of Obstetrics and Gynaecology when the deaths occurred. Information was extracted from the case files at the end of 2007. Data relating to the total number of deliveries were obtained from the registers kept in the labour and isolation wards.

**Results:**

Of the 45 maternal deaths recorded, 40 (88.9%) were found to have occurred among the unbooked and 5 (11%) among the booked mothers, constituting a maternal mortality ratio (MMR) of 23 121.4 and 339.7 per 100 000 deliveries respectively. The combined mortality ratio was 2 735.6 per 100 000 deliveries. Fifteen (37.5%) unbooked primigravidae were found to have died of severe pre-eclampsia/eclampsia. A total of 1 645 mothers were noted to have delivered babies, of whom 1 472(89.5%) were booked, and 173 (10.5%) unbooked, with the hospital.

**Conclusion:**

Severe pre-eclampsia/eclampsia, haemorrhaging and sepsis were the major causes of death. A high maternal mortality rate was found to be common among the unbooked primigravidae, who are known usually to present late with pre-eclampsia/eclampsia. More research into the causes and management of pre-eclampsia/eclampsia is needed to reduce the high maternal death rate associated with it. The lack of antenatal care is also a high risk factor for maternal death.

## INTRODUCTION

At the close of the last century, sub-Saharan Africa still experienced high maternal mortality rates, with the goal of the provision of conditions conducive to safe motherhood eluding many governments. For many years, in spite of the efforts exerted in this regard, the evidence shows that the rate of maternal deaths was on the rise. The rate of such deaths in Nigeria is the second highest in the world.^1^ Beginning with the launch in 1987 of the Safe Motherhood Initiative in Nairobi, Kenya, a decline in the rate of such deaths was anticipated. Unfortunately, many of the good intentions of the initiative failed to materialise. The maternal death rate in the rural areas of Nigeria is rapidly increasing, due to the extreme poverty experienced in the area.^2^ Maternal deaths in the developing countries has been described as comprising a multitude of quiet tragedies and a disgrace to the modern world.^3, 4^ The estimated maternal death ratio of 1 000 to 1 500 deaths per 100 000 deliveries in Nigeria is high, considering the fact that the maternal mortality rate in developed countries is less than 100 per 100 000 deliveries, with only 25% of women of reproductive age living in such countries. The maternal mortality statistics provide one of the worst differentials in health indices in respect of the developed and developing countries. For example, Africa had 30% of maternal deaths worldwide in 2005, and only 11% of women live in Africa The risk of dying from pregnancy-related causes is 1 in 1 750 in the developed countries, 1 in 870 in East Asia, 1 in 90 in Latin America and 1 in 24 in Africa.^5^ If the maternal mortality ratio (MMR) were the same in the developing countries as that in the developed countries, 360 000 fewer women would die each year.

The global objective of reducing maternal mortality by at least 50% by the year 2000 clearly was not attained. The rate of maternal mortality has, instead, increased. Redressing gender-based social inequities, ensuring that couples have access to family planning, improving community-based maternity care and providing backup and support at the first referral level for women who require skilled obstetric care are all strategies that have been identified as means of reducing maternal death.^5^ Such goals have yet to be met. Most (80% +)^5^ deaths that occur in Nigeria are due to obstetric haemorrhaging, sepsis and eclampsia, resulting, most notably, in the need for effective strategies to prevent and cure pre-eclampsia/ eclampsia. In the current study, the unacceptably high rate of maternal mortality resulting from severe pre-eclampsia/eclampsia in Nigeria is highlighted. The conditions leading to unbooked emergencies also require urgent redress.

## METHOD

The data from the case notes taken on all deaths, resulting from both obstetric and gynaecological causes, which occurred in the Department of Obstetrics and Gynaecology at Ebonyi State University Teaching Hospital, Abakaliki (EBSUTHAI) for the year ending 31 December 2007 were collected and used for the current study. All deaths that occurred in the unit between 1 January and 31 December 2007 were reported to the Head of the Department of Obstetrics and Gynaecology, who stored the relevant case files until the end of the year. The details relating to the distribution of deaths, according to booking status, the clinical causes of death and the parity of the patients, were then extracted from the case files for purposes of analysis.

The total number of deliveries was obtained from the register kept in the isolation ward for the unbooked patients, as well as from the register kept in the main labour ward for the booked patients.

## RESULTS

The total number of deliveries during the 12 months covered by the current study was 1 645. Of this number, 1 472 (89.5%) received antenatal care in EBSUTHAI, while 173 (10.5%) did not. A total of 45 deaths was found to have occurred, with five (11.1%) of the deaths occurring among the booked women and 40 (88.9%) among the unbooked women. The MMR was 2 735.6 per 100 000 deliveries, with it being 339.7 per 100 000 deliveries for the booked women and 23 121.4 per 100 000 deliveries for the unbooked women. In other words, for every 100 000 deliveries, the figures represent the MMR as shown above (see Table 1).

**TABLE 1:** Distribution of deaths, according to booking status

	NO. OF DEATHS OF MOTHERS	PERCENTAGE (%)	TOTAL DELIVERIES	PERCENTAGE (%)	MMR/100 000 DELIVERIES
Booked	5	11.1	1 472	89.5	339.7
Unbooked	40	88.9	174	10.5	23 121.4

**TOTAL**	**45**	**100**	**1 646**	**100**	**23 461.1**

Table 2 shows the obstetric and medical causes of the maternal deaths. Severe pre-eclampsia/eclampsia accounted for 35.6%, and obstetric haemorrhaging 28.9% of the deaths. Sepsis and anaesthesia accounted for 13.3% and 8.9% of the deaths respectively. More than one obstetric and medical factor are usually present simultaneously in the case of the death of a patient, with patients with severe pre-eclampsia/eclampsia also tending to have sepsis, haemorrhaging or acute renal failure.

**TABLE 2:** Obstetric and medical causes of deaths

CAUSE OF DEATH	BOOKED	PERCENTAGE (%)	UNBOOKED	PERCENTAGE (%)	TOTAL	PERCENTAGE (%)
Severe pre-eclampsia/eclampsia	1	20	15	37.5	16	35.6
Haemorrhage	3	60	10	25.0	13	28.9
Sepsis	1	20	5	12.5	6	13.3
Anaesthesia	-	-	4	10.0	4	8.9
Viral hepatitis	-	-	3	7.5	3	6.7
HIV/AIDS	-	-	1	2.5	1	2.2
Induced abortion and its complications	-	-	2	5.0	2	4.4

As shown in Table 3, the number of maternal deaths of both booked and unbooked mothers, except for the parity of 3, is striking in nulliparous patients. In 12 of the cases, no record was made of the patients’ parity.

**TABLE 3:** Effect of parity on maternal deaths

PARITY	BOOKED	PERCENTAGE (%)	UNBOOKED	PERCENTAGE (%)	TOTAL	PERCENTAGE (%)
0	–	–	15	37.5	15	33.3
1	2	40	5	12.5	7	15.6
2	3	60	2	5.0	5	11.1
3	–	–	–	–	–	–
4	–	–	3	7.5	3	6.7
5	–	–	2	5.0	2	4.4
> 5	–	–	1	2.5	1	2.2
Not stated	–	–	12	30.0	12	26.7

**TOTAL**	**5**	**100**	**40**	**100**	**45**	**100**

## DISCUSSION

The data relating to maternal deaths as discussed in the current study are some of the highest in the world. Harrison^7^ revealed how a maternal mortality rate of 24 per 1 000 deliveries still exists in a remote part of rural West Africa. The current situation has shown an even more marked deterioration in the area, as well as in the rice-producing city of Abakaliki in the Ebonyi State of Nigeria.

Over a period of 13 years at the University of Benin Teaching Hospital, 29 324 deliveries were recorded as having taken place, with a maternal mortality rate of 536 per 100 000 deliveries.^10^ While one death occurred among the booked patients for every 294 deliveries, one occurred among the unbooked patients for every four deliveries. The phenomenon of unbooked patients persists in Nigerian society, due to the prevailing social, economic, cultural and political conditions that, in the first place, led to such a phenomenon. The MMR among unbooked patients should be reduced by their being treated promptly on their arrival at the hospital, which should help to prevent further deterioration of their already precarious state of health. Such treatment entails the provision of consumables, such as antibiotics, intravenous fluids, drip sets, stored blood, and cannulae. Emergency operative deliveries should be carried out directly after the initial resuscitative measures have been applied. Most of the requirements for medical intervention in the case of such patients is currently provided by their relatives, so that the majority of patients, who cannot afford to pay for such intervention, die. The leading medical causes of obstetric deaths among the patients covered by the current study were found to be severe pre-eclampsia/eclampsia, haemorrhaging and sepsis. Acute renal failure was also found to result from severe pre-eclampsia/eclampsia. In the developed countries, including those in Europe, eclampsia has been found to complicate about 1 in 2 000 deliveries, while, in the developing countries, the estimates of such complications vary widely from 1 in 100 to 1 in 1 700.^12, 14^ Though the late presentation of the eclamptic patients accounted for the high MMR observed, the correct management of the disease is largely unknown. Most of the causes of maternal deaths are preventable, but those of pre-eclampsia, which progresses to eclampsia, have continued to be enigmatic, with the treatment being empirical. Consequently, more research into the aetiology, pathophysiology and effective management of pre-eclampsia/eclampsia is urgently needed to avoid the high MMR associated with the disease entity concerned, as found in the current research.

Early writers on maternal deaths in Nigeria have highlighted the high rate of maternal mortality associated with high parity.^15, 16^ However, the current study shows that the higher number of maternal deaths was found to occur in the primigravidae, while the figure was relatively low among the grand multiparous women. The fact that severe pre-eclampsia/eclampsia is more common among the primigravidae would have accounted for the high number of maternal deaths among the subjects of the current study.

### Conclusion

The causes of maternal death are usually multifactorial. However, the current study emphasises how the lack of antenatal care can result in the development of severe pre-eclampsia/ eclampsia. More research into the causes and management of pre-eclampsia/eclampsia is, therefore, needed to reduce the high rate of maternal mortality associated with it. Primigravidae who are prone to such an illness should be encouraged to book in for antenatal care, which should help to ensure the early detection of pre-eclampsia, allowing for the appropriate treatment to be applied. The importance of maintaining a ready supply of stored blood in the blood banks for use in such emergencies cannot be over-emphasised.
